# Clinical evaluation of [^123^I]FP-CIT SPECT scans on the novel brain-dedicated InSPira HD SPECT system: a head-to-head comparison

**DOI:** 10.1186/s13550-018-0436-y

**Published:** 2018-08-22

**Authors:** Sofie M. Adriaanse, Tim C. de Wit, Mette Stam, Eline Verwer, Kora M. de Bruin, Jan Booij

**Affiliations:** Department of Radiology and Nuclear Medicine, Academic Medical Center, Amsterdam, The Netherlands

## Abstract

The InSPira HD system, a novel brain-dedicated SPECT scanner, allows for imaging with a high spatial resolution. Here, we tested whether this scanner can be used to image the dopamine transporter adequately. Therefore, striatal phantom and patient data acquired on the InSPira were compared head-to-head with the well-validated brain-dedicated NeuroFocus system. A striatal phantom filled with [123I] and 14 subjects (after [123I]FP-CIT injection) were scanned on both systems. [123I]FP-CIT SPECT scans were visually assessed. Striatal binding ratios were calculated automatically using the software package BRASS. Striatal phantom and patient data showed strong correlations with respect to striatal ratios (*R* = 0.99 and *R* = 0.92; *p* < 0.05 and *p* < 0.01, respectively). Slightly higher ratios were found for the NeuroFocus patient data, probably due to differences in system performance. Visual assessment of [123I]FP-CIT scans showed agreement between systems in 13 of the 14 cases. We conclude that [123I]FP-CIT SPECT imaging can be performed adequately on the new InSPira system.

## Rationale

The InSPira HD system (InSPira; Neurologica, Danvers, MA, USA) is a novel brain-dedicated single-photon emission computed tomography (SPECT) system which differs from conventional SPECT scanners with respect to hardware configuration and scanning geometry [1]. Performance of the InSPira system was investigated extensively using phantom data by our group [2]. Good spatial resolution was found, which was 3 mm in air, in the center of the field of view (FOV). In comparison, conventional SPECT systems have a spatial resolution of ~ 7 to 12 mm [3].

In routine practice, the majority of neuro SPECT studies within the Academic Medical Center consist of dopamine transporter (DAT) SPECT imaging [4–6]. DAT imaging with [^123^I]FP-CIT is an established diagnostic tool to detect loss of the dopaminergic nigrostriata pathway, a hallmark of Parkinson’s disease (PD). PD patients show reduced striatal DAT binding, particularly in the putamen [3, 7]. [^123^I]FP-CIT SPECT images at our department were routinely performed on the brain-dedicated NeuroFocus system (Medfield, MA, USA). This system was installed in our hospital in 1990 and recently replaced by the InSPira system. Our aim was to investigate whether this new system would provide similar results, with respect to DAT imaging, as acquired on the NeuroFocus. For this, a striatal phantom and subjects were scanned on both SPECT systems.

## Methods

### Patients

All procedures performed in studies involving human participants were in accordance with the ethical standards of the Academic Medical Center medical ethical committee and with the 1964 Helsinki declaration and its later amendments or comparable ethical standards. Informed consent was obtained from all individual participants included in the study. Subjects were scheduled for a routine DAT SPECT scan on the NeuroFocus for clinical evaluation of possible neurodegenerative parkinsonism or participated in a research project on heroin addiction (*n* = 2; these participants had a 2-week heroin- and methadone-free period between the end of detoxification and the SPECT scan. All subjects were required to have a negative urine drug screen for opioids, cocaine, and amphetamine on the day of the SPECT scan) in which the striatal DAT binding was examined [6]. A total of 24 subjects agreed to participate. They were injected with ~ 111 MBq ^123^I-ioflupane ([^123^I]FP-CIT; GE Healthcare) and scanned on the NeuroFocus 3-h post-injection [6]. Patients were scanned directly after finalization of the NeuroFocus scan, on the InSPira system (~ 4-h post-injection). Subjects did not use any medication known to influence [^123^I]FP-CIT binding. Data from seven subjects was excluded because of early termination of the scan on the InSPira system because of fatigue of the subjects due to the antecedent scan obtained on the NeuroFocus. In addition, for two patients, technical problems during the scan on the InSPira resulted in inability to reconstruct the data. For one patient, technical problems on the NeuroFocus resulted in insufficient quality for quantification, and the patient was therefore excluded. Consequently, complete data sets for 14 subjects were available for analysis.

### Striatal phantom

The striatal phantom (RS-901T, Radiology Support Devices Inc., CA, USA) consists of left and right caudate nucleus, left and right putamen, and the rest of the brain. Striatal compartments were filled with 30/40 kBq/mL iodine-123 (^123^I) (left/right), and the rest of the brain with a range of activity concentrations, 5, 10, and 15 kBq/mL ^123^I, as earlier described [3].

### NeuroFocus scan procedure

For details on the NeuroFocus system, see [5, 6]. In short, the NeuroFocus system has 12 individual crystals equipped with a focusing collimator (resulting in sensitivity being higher in the central focal point), and a spatial resolution of approximately 6.5 mm FWHM throughout the 20-cm FOV. With a bore diameter of 28.6 cm. Axial slices were acquired, parallel and upward from the orbitomeatal line at least covering the entire striatum. For the phantom scans, a slice time of 180 s and a slice spacing of 4 mm was used. The energy window was set at 159 keV, with 20% lower and upper boundaries (resulting in an energy window of 143–175 keV). For subjects, slice thickness of 10 mm, slice spacing of 5 mm, and slice timing of 150 s were applied (slice timing of 180 s was applied for subjects that were part of the research project) [6]. A proprietary iterative reconstruction algorithm was used to reconstruct the data (with a fixed, unknown, number of iterations). Attenuation correction for patient data was performed using a spherical volume (with a uniform attenuation coefficient of 0.0105/mm) that was manually aligned using a rigid transformation.

### InSPira scan procedure

For details on the InSPira system, see [1, 2]. In short, high-resolution imaging is achieved by the unique design of the detector ring of the InSPira HD. The detector ring consists of two clamshells, each containing 12 (focusing) fanbeam collimators, creating a 29.0-cm diameter bore. At start position, the two clams touch and the collimators are focused at the center of the ring, achieving a focal point of 3 mm in diameter. During acquisition, the gantry rotates and simultaneously the two clams are moved outward leading the two focal points of the clam’s collimators to follow spiral trajectories over the field of view (FOV). A proprietary iterative reconstruction algorithm (with 60 iterations) tailored to this unique method of spatial sampling is used to reconstruct the data into 3D images. This iterative reconstruction algorithm is based on a maximum a posteriori (MAP) estimation. It includes a point spread function (PSF), which is defined as the detector response to an impulse activity source point placed in the scanner FOV [2]. For positioning see section “NeuroFocus scan procedure”. For phantom studies, the same scan parameters were applied as on the NeuroFocus. A CT-scan of the phantom was used for attenuation correction. For subjects, slice timing ranged from 120 to 240 s, slice thickness from 3.125–6 mm. The energy window was set at 159 keV, with 20% lower and upper boundaries (resulting in an energy window of 143–175 keV). An adult head CT template was manually aligned and used for attenuation correction.

### Visual assessment

All scans were visually evaluated by an experienced reader (JB), who was blinded to clinical data and scanner. Striatal binding was rated as normal when bilateral caudate nucleus and putamen showed high and symmetric [^123^I]FP-CIT binding [8]. If the loss of DAT binding was more pronounced in the putamen than in the caudate nucleus on one or both sides, the scan was rated as abnormal supporting the clinical diagnosis of PD.

### Striatal quantification

The Brain Registration & Analysis Software Suite (BRASS™, HERMES Medical, Sweden) was used for phantom and patient data analysis to quantify striatal DAT binding, as earlier described [9]. BRASS automatically registers the patient data to a template containing a number of VOIs and calculates bilateral caudate nucleus, putamen, and whole striatal binding ratios. This was done by subtracting counts in the VOIs with counts in the reference region (occipital cortex) and finally dividing by counts in the reference region (i.e., specific striatal to non-specific binding ratios, which reflects the binding potential). The ENC-DAT control database [3] as implemented in BRASS was used as a reference template and for region definition. An independent sample of healthy subjects scanned on the InSPira was used to assess if patient’s striatal ratios fell within the normal range (only available for InSPira data) [4].

### Statistical analysis

Pearson correlations were calculated to assess associations between striatal ratios of the two systems (the average of left and right striatal ratios). In order to obtain more detailed insight into the agreement between the two systems, Bland-Altman plots were examined. Limits of agreement were set at ± 1.96 standard deviation from the mean difference.

## Results

### Visual assessment

**Fig. 1:**
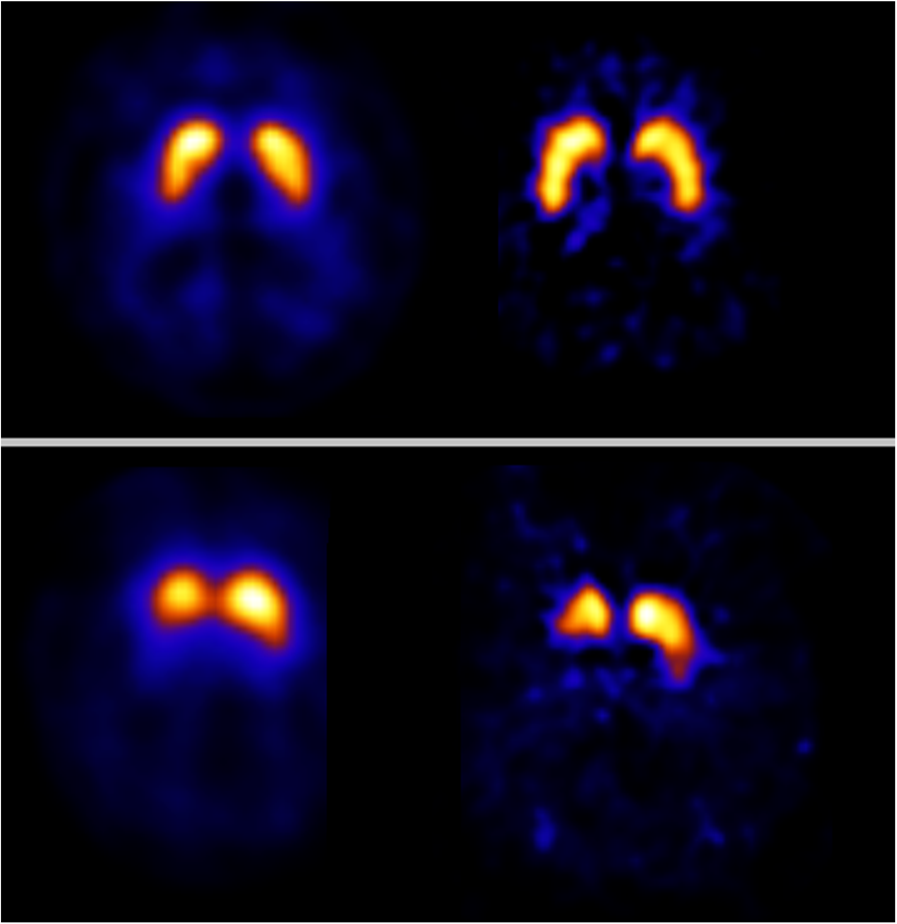
[^123^I]FP-CIT SPECT scans acquired on the NeuroFocus and InSPira. Representative image of a [^123^I]FP-CIT SPECT scan rated as “normal” (upper panel), and as abnormal (PD-like) (lower panel) on both systems. Images on the left and right were acquired on the NeuroFocus and InSPira, respectively. Images are displayed in radiological orientation (left is right)

**Fig. 2:**
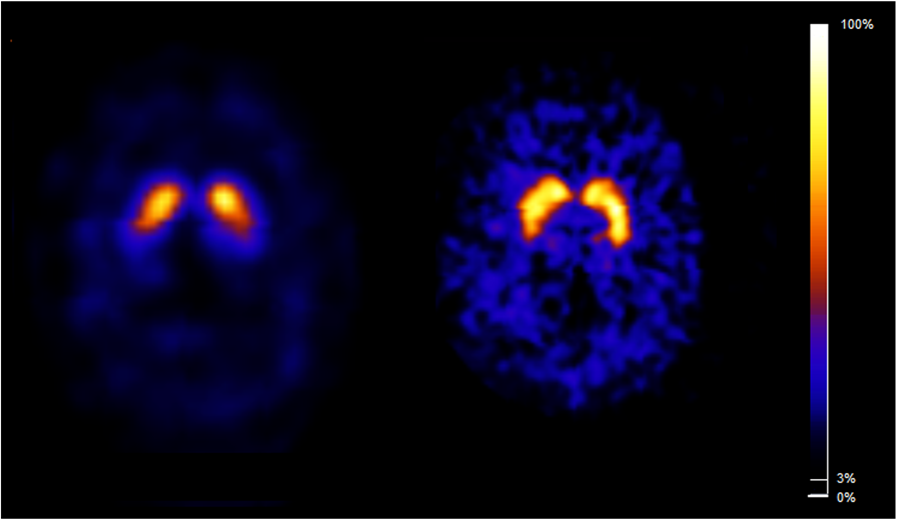
Discordant visual rating between both systems. Tranversal images of the [^123^I]FP-CIT SPECT scans rated visually as abnormal (PD-like; due to relatively low binding in the left putamen vs left caudate nucleus) on the NeuroFocus (left panel) and as “normal” on the InSPira (right panel) obtained in subject #8. Images are displayed in radiological orientation (left is right)

Visual assessment of patient data showed agreement for 13 of 14 scans for both systems (7 normal; 6 abnormal—PD-like). See Fig. 1 for an example of a normal and abnormal scan rated equally on both systems. Disagreement was found for one scan, which was interpreted visually as normal on the InSPira, and as abnormal (PD-like) on the NeuroFocus (Fig. 2). Interestingly, the quantification showed that the specific to non-specific striatal [^123^I]FP-CIT binding ratio of this subject was within the low normal range for the InSPira system (see “Discussion”).

### Striatal quantification

Registration of phantom and patient data to the template using BRASS was successful for all included scans.

**Fig. 3:**
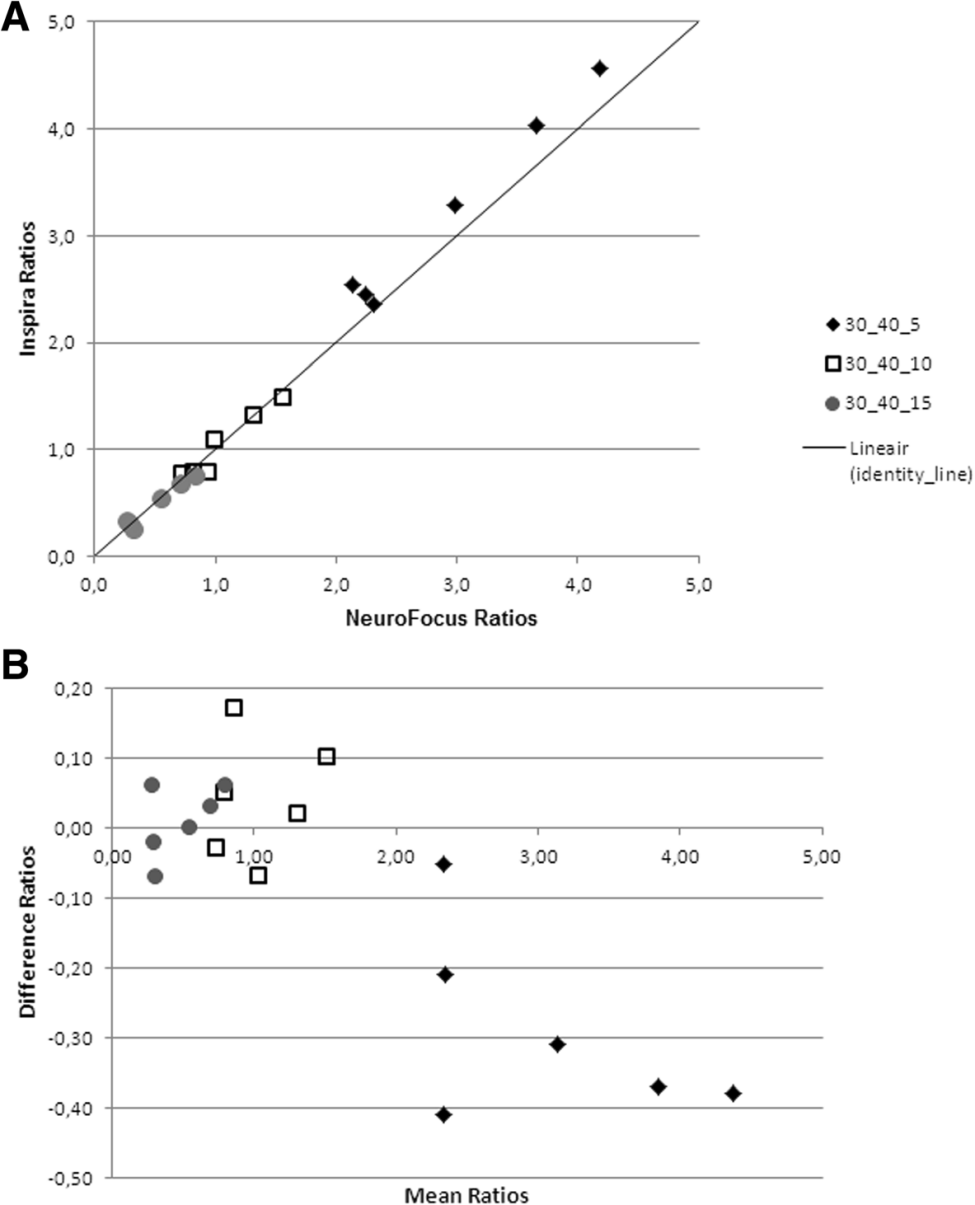
Scatterplot of [^123^I]FP-CIT binding ratios in the caudate nucleus and putamen on the InSPira and NeuroFocus system in phantom data. **a** Scatterplot of specific to non-specific [^123^I]FP-CIT binding ratios in the caudate nucleus and putamen of data obtained on the InSPira (*y*-axis) and NeuroFocus (*x*-axis) system for the striatal phantom. **b** Bland-Altman plot with mean ratios on the two systems on the *x*-axis and the differences between the two systems on the *y*-axis (subtracting InSPira from NeuroFocus values). All data fell between the limits of agreement (± 1.96 SD; not displayed)

Specific to non-specific striatal binding ratios showed good correlations between both systems for phantom data, with Pearson’s *R* = 0.9993 (*p* = 0.02; Fig. 3a). The Bland-Altman plot (Fig. 3b) revealed that all data points fell between the limits of agreement.

**Fig. 4:**
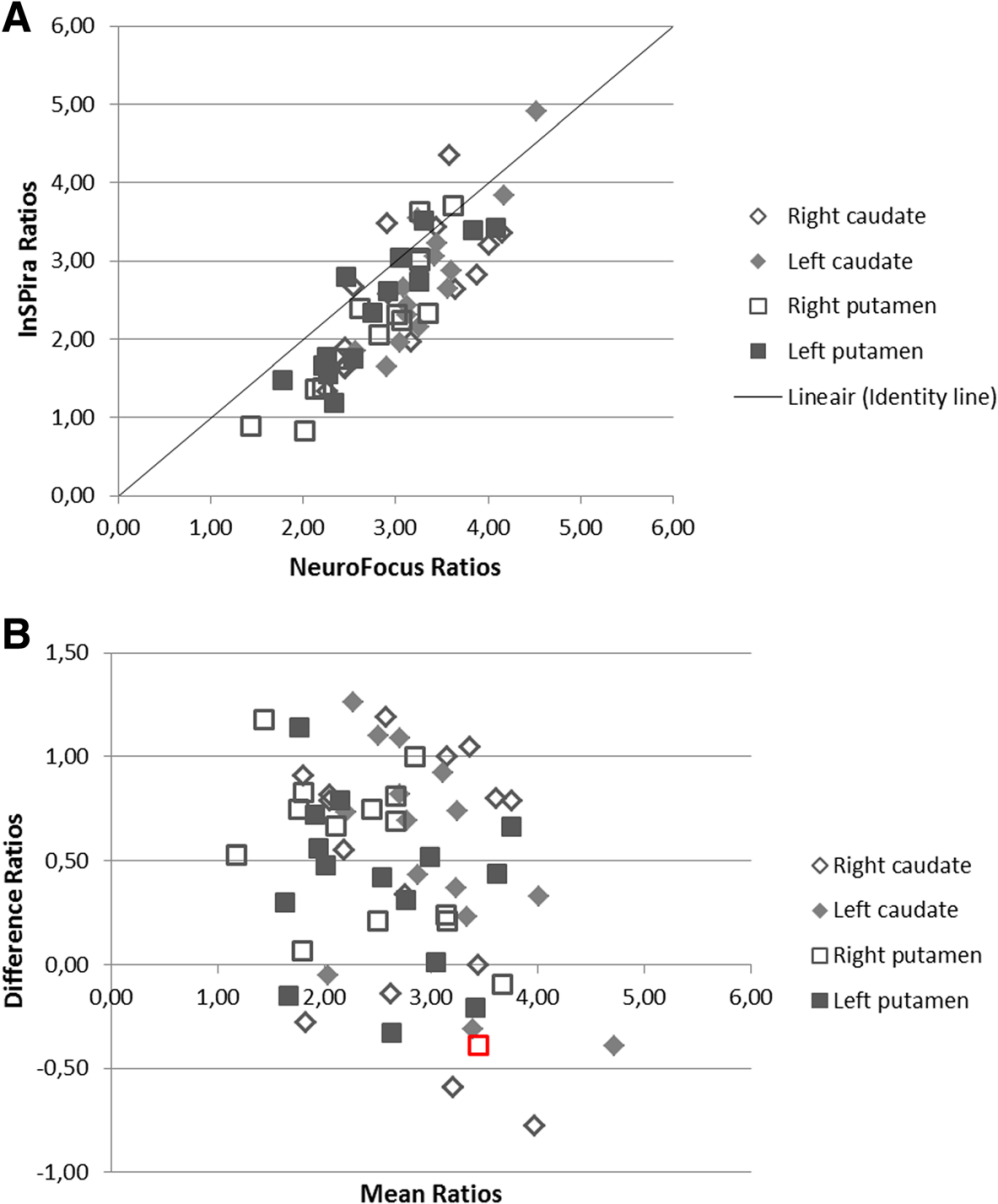
Scatterplot of [^123^I]FP-CIT binding ratios in the caudate nucleus and putamen on the InSPira and NeuroFocus system in patient data. **a** Scatterplot of specific to non-specific [^123^I]FP-CIT binding ratios in the caudate nucleus and putamen of data obtained on the InSPira (*y*-axis) and NeuroFocus (*x*-axis) system for the human study. **b** Bland-Altman plot with mean ratios for the two systems on the *x*-axis and the differences between the two systems on the *y*-axis (subtracting InSPira from NeuroFocus ratios). The values exceeding 1.96 SD difference (for that ROI) are displayed in red. One value exceeded our pre-defined limits of agreement (1.96 SD difference). This was right putamen ratio for a subject whose [^123^I]FP-CIT SPECT scan was visually rated as abnormal (PD-like) on both systems

With respect to specific to non-specific striatal [^123^I]FP-CIT binding ratios in the human study, the correlation analysis showed a relatively good association between both systems (Fig. 4a; *R* = 0.92; *p* < 0.01). In general, the striatal [^123^I]FP-CIT ratios acquired on the NeuroFocus system were higher (~ 20%) compared to the InSPira, which can also be observed from the scatter and Bland-Altman plots (Table 1; Fig. 4a, b). This effect seemed to become less apparent for high ratios. One data point fell outside the limits of agreement.

**Table 1:** Mean specific to non-specific [^123^I]FP-CIT binding ratios (± SD) in bilateral caudate nucleus and putamen of data obtained on the NeuroFocus and InSPira system in 14 subjects

	Right caudate	Right putamen	Left caudate	Left putamen
NeuroFocus	3.13 (± 0.65)	2.74 (± 0.63)	3.36 (± 0.50)	2.79 (± 0.66)
InSPira	2.65 (± 0.87)	2.21 (± 0.92)	2.79 (± 0.88)	2.37 (± 0.88)

## Discussion

### Visual assessment

The InSPira system has the advantage of a high spatial resolution [1, 2]. When examining [^123^I]FP-CIT SPECT images from the InSPira system, the spatial resolution in comparison to the NeuroFocus system is indeed higher. Visual assessment of DAT scans was in agreement between both systems; visual interpretation disagreed for only one single case. The time delay between the two scans could have resulted in differences in striatal binding/wash out [3, 7]. It is however unlikely that this small effect explains the disagreement in visual assessment for the single subject, whom was part of a study examining heroin addiction. Heroin addicts are known to have somewhat lower striatal [^123^I]FP-CIT binding ratios than age-matched healthy controls [6, 10], making visual interpretation more difficult. It was expected that visual inspection of DAT SPECT scans in abstinent heroin-dependent subjects would reveal a normal distribution within striatal regions, since these subjects are not reported to have clinical symptoms associated with neurodegeneration. Lower striatal [^123^I]FP-CIT binding ratios are reasoned to be an effect of addiction in general. Indeed, quantification revealed relatively low striatal binding ratios in this subject for the InSPira scan, which still fell within the normal range using an age-matched control database. And, the visual rating based on the InSPira scan was most in line with expected results. The reason for the discordance between the two systems is not straightforward. It could be due to differences in system performance (see also below) or due to poorer resolution for the NeuroFocus. Most likely, visual interpretation for cases with relatively low striatal [^123^I]FP-CIT binding ratios may be challenging. This emphasizes the need for quantification in combination with visual assessment in routine practice.

### System performance and its effect on striatal quantification

The most striking observation was that human specific to non-specific striatal [^123^I]FP-CIT binding ratios on the NeuroFocus system were found to be slightly higher (~ 20%) compared to the InSPira. This was not observed in the phantom study. In general, differences up to 30% between cameras are not uncommon [3] and can be even greater when processing steps differ. It is unlikely that these differences were (partly) caused by binding/wash-out differences 3- and 4-h post-injection, since an opposite effect would be expected; slightly *higher* specific to non-specific striatal [^123^I]FP-CIT binding ratios in healthy controls were found at 4 h compared to 3 h post-injection in a multi-center study [3]. It is therefore probable that these differences are caused by system performance. Unfortunately, the replacement of the NeuroFocus was scheduled with the arrival of the InSPira and was dismantled. For the acquisition of the phantom and patient scans presented in this paper, there was limited time where the two systems were both available. Therefore, we were not able to examine extensively and directly the difference in system performance and acquisition/processing differences between both systems. In a previous paper from our group, however, the technical performance of the InSPira system was described [2]. This paper reported on scanner performance characteristics, such as spatial resolution and recovery. Since recovery was very similar between the two systems, it is not likely that this caused the observed differences. It should be noted that no correction for resolution recovery was applied for either systems. Another difference between the systems is that the reconstruction algorithm of the NeuroFocus corrects for background count rate by default. Unfortunately we do not have detailed insights into how the reconstruction algorithm incorporates this background rate and can therefore only hypothesize about possible effects. Subtraction of a certain constant value is expected to artificially enhance striatal ratios, particularly high ratios. Indeed, it was observed that increasing background count rate in the reconstruction for NeuroFocus phantom data was in line with this hypothesis, but only accounted for a few percent difference. Therefore, we believe that this might only partly explain the higher ratios observed on the NeuroFocus compared to the InSPira. Perhaps most important, the collimation of the two systems could have an impact on the image quality; scatter and energy penetration could affect quantitation. Interestingly, for clinical data strongest differences were observed. These effects were not so apparent for the phantom data. This seems to indicate that activity outside the field of view, resulting from activity distribution in the body of the participants, may explain differences between the systems. Indeed, a comparatively higher background was observed for the InSPira patient data, when compared to the NeuroFocus patient data. It may therefore be hypothesized that the InSPira may be more affected by septal penetration than the NeuroFocus, which may explain the lower striatal binding ratios observed for the Inspira system in the patient studies. It should be noted that no correction for scatter was performed for either of the systems. Finally, acquisition and processing differences between the systems, such as smoothing parameters, slice thickness, and resolution, could have influenced quantification by affecting automatic registration in BRASS. However, we feel that this does not likely explain the quantitative differences, since visual inspection of registration of phantom and patient data to the template using BRASS seemed successful for scans of both systems.

We are confident that although these differences between the systems exist, this will not affect diagnostic accuracy when visual assessment of DAT SPECT images is done together with quantitative assessment using an appropriate reference database for that specific system.

### Limitations

A limitation of the present study was the difference in scan time between the two systems, because the patients were referred for routine clinical studies, or participated in a study, in which they should receive optimal usual medical care with standardized acquisitions times (i.e., imaging 3-h post-injection of [^123^I]FP-CIT on the standard (NeuroFocus) system). In addition, data from a relatively large number of subjects was unsuitable for analysis, which were mostly PD patients, and thus might have resulted in an underrepresentation of abnormal (low) striatal binding ratios. Finally, no extra phantom experiments could be performed to assess whether differences in system performance resulted in the quantitative findings between the two systems.

## Conclusion

In conclusion, [^123^I]FP-CIT SPECT imaging can be performed adequately on the new InSPira system.
